# Data supporting the evaluation of the energy recovery potential of thermoelectric generators in diesel engines

**DOI:** 10.1016/j.dib.2019.105075

**Published:** 2019-12-30

**Authors:** Rafael Ramírez, Alexis Sagastume Gutiérrez, Juan J. Cabello Eras, Brando Hernández, Jorge Duarte Forero

**Affiliations:** a Universidad de la Costa CUC, Calle 58 Número 55 - 66, Barranquilla, Colombia; b Universidad del Atlántico, Department of Mechanical Engineering, Carrera 30 Número 8 - 49, Puerto Colombia, Área Metropolitana de Barranquilla, Colombia

**Keywords:** Thermoelectric generator, Thermoelectric module, Heat exchanger, Internal combustion engine

## Abstract

Power generation with thermoelectric devices in internal combustion engines is an alternative to recover some of the energy loss with the exhausts. This data article supports a study that assesses the potentialities of energy recovery with thermoelectric generators in diesel engines and its influence on gaseous emissions. To this end, a set of experiments was developed with a thermoelectric generator and a waffle heat exchanger. The experimental design included nine operation points of the engine to characterize the energy recovery of the thermoelectric generator under different exploitation conditions. Three different fuels (i.e., diesel, B5, and B10) were used. The experiments were developed in a test bench with three data acquisition systems to measure the operational variables (e.g., electric power generation, pressure drop, temperature, etc.). Moreover, a gas analyzer (BrainBee AGS-688), Bacharach gas analyzer (PCA 400), and a smoke meter (BrainBee OPA-100) were used to measure exhaust emissions.

**Table:** Specifications Table

Subject	Energy Engineering and Power Technology
Specific subject area	Heat transfer in heat exchangers and power generation in thermoelectric generators
Type of data	Table Figure
How data were acquired	The experiments were developed in SOKAN- SK-MDF300 diesel engine on a test bench. The test bench includes three data acquisition systems to measure the engine parameters, the temperatures in the thermoelectric modules (TEMs) were measured using type k thermocouples, while the pressure drop was measured with a PSA-CO1 pressure sensor. In addition, the engine emissions (i.e., CO_2_, NO_X_, smoke opacity, etc.) were measured with a BrainBee AGS-688 gas analyzer, Bacharach PCA 400 gas analyzer, and a BrainBee OPA-100 smoke meter.
Data format	Raw
Parameters for data collection	Nine operating points of a diesel engine were considered in the experiments. The operation points included torques from 3 Nm to 6 Nm and rotation speeds from 3000 rpm to 3800 rpm. Three fuels, including diesel, B5, and B10, were used during the experiments.
Description of data collection	The experimental design was developed using the STATGRAPHICS Centurion XVI, with a multilevel factorial experimental design 3^3^, including the three variable levels. The measures on each operating point were repeated three times.
Data source location	Laboratory of Thermal Machines, Universidad del Atlántico. Barranquilla, Colombia.
Data accessibility	Within the article
Related research article	[1] Ramírez, R., Gutiérrez, A., Cabello Eras, J., Valencia, K., Hernández, B., Duarte, J., 2019. “Evaluation of the energy recovery potential of thermoelectric generators in diesel engines,” J. Clean. Prod. 241, 118412. DOI: 10.1016/j.jclepro.2019.118412.

**Table:** 

**Value of the Data** • The data shows the influence of different operating conditions in a diesel engine in the performance of a thermoelectric generator, identifying which of these conditions offers better performance with the heat exchanger used. • The data shows the influence of the diesel – biodiesel fuel blends on the performance of thermoelectric generator operation and its influence on engine emissions. • This data can be used as a benchmark for researchers to assess the improvements in the efficiency of thermoelectric generators in diesel engines. • This data can be used as a guide to assess the performance of thermoelectric generators in gasoline engines. • This data can be used to assess the influence of a thermoelectric generator in the energy efficiency of diesel engines. • This data can be used to assess the influence of a thermoelectric generator in the thermal and environmental performance of a diesel engine.

## 1 Data

**Fig. 1:**
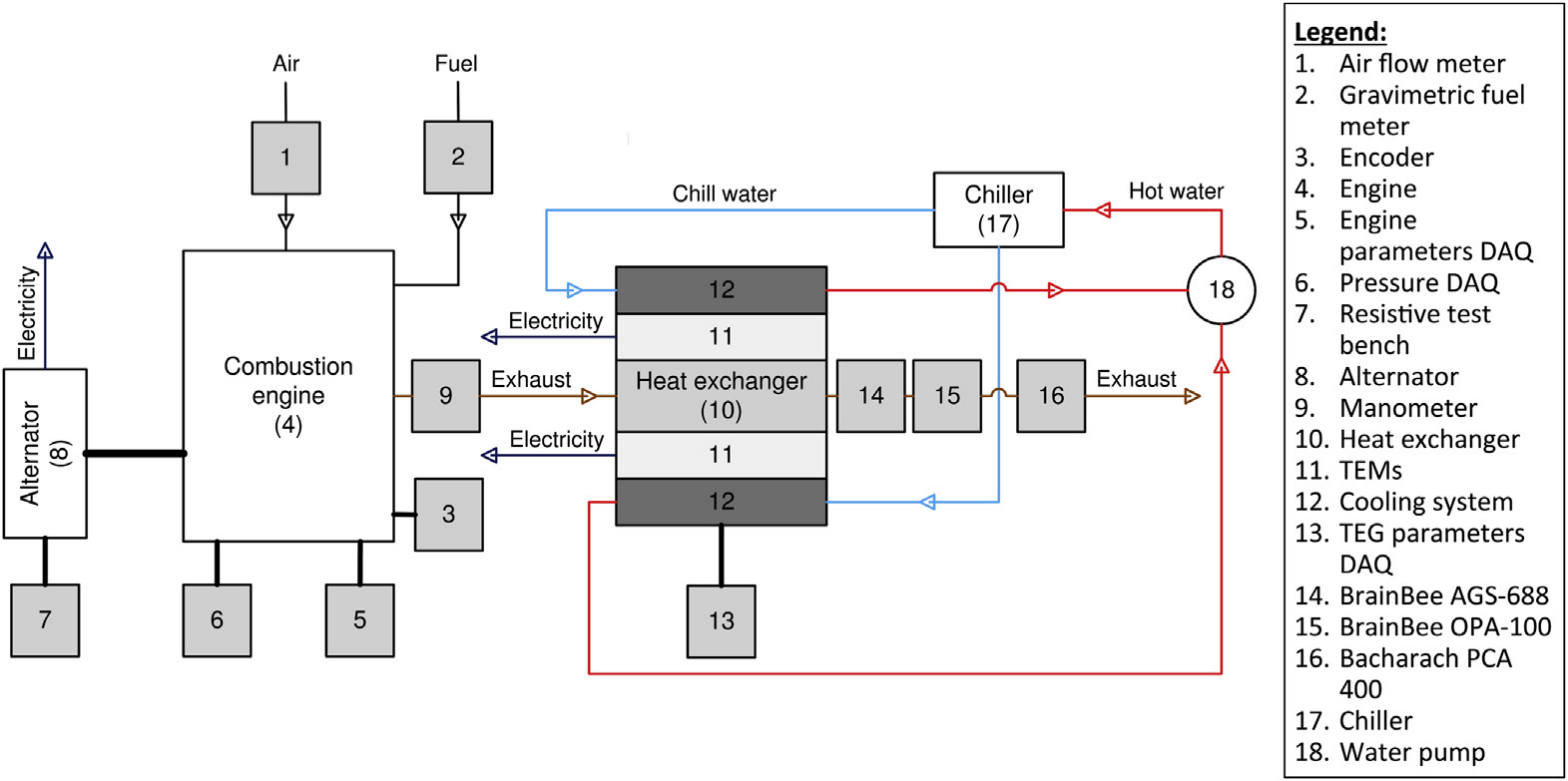
Schematic diagram of the experimental facilities.

The data presented correspond to the experiments developed to assess the energy recovery potential of a thermoelectric generator (TEG) in diesel engines. The test bench used for the experimentation is shown in Fig. 1. Table 1 shows the temperature of the thermoelectric modules (TEMs) measured during the use of B5 (95% diesel+5% biodiesel) in the diesel engine. Furthermore, Table 2 shows the temperature of the TEMs measured during the use of B10 (90% diesel + 10% biodiesel) in the diesel engine. Similarly, Table 3 shows the temperature of the TEMs measured during the use of diesel in the engine. Moreover, Table 4 shows the pressure drop measured in the TEG during the experiments for the different operation points and the different fuels.

**Table 1:** Temperature of the TEMs during the combustion of B5.

Operation point	Test	Temperature (°C)									
		1	2	3	4	5	6	7	8	9	10
A	1	121.43	115.14	113.78	114.97	110.43	110.23	108.45	105.56	100.00	98.43
	2	123.43	113.15	112.98	112.76	108.65	108.54	106.76	105.53	101.12	96.75
	3	121.89	114.19	115.30	113.46	109.15	109.04	106.99	109.79	104.88	98.10
B	1	153.54	135.23	136.21	136.32	131.53	131.84	127.43	127.21	123.38	113.87
	2	155.21	135.23	135.87	135.11	130.15	128.76	126.65	125.54	121.54	115.53
	3	151.60	138.08	136.04	135.82	128.29	128.53	126.41	127.29	123.90	114.82
C	1	176.45	163.20	161.23	158.85	155.12	152.64	150.53	146.76	142.43	135.75
	2	177.76	164.34	160.30	160.78	153.75	154.32	148.54	148.98	138.85	133.86
	3	180.09	161.76	162.58	162.77	153.64	152.10	150.51	145.68	140.28	133.86
D	1	187.38	171.21	167.32	165.27	162.25	160.05	154.36	155.36	150.47	136.54
	2	188.73	167.45	168.94	166.46	160.05	161.32	156.32	153.15	152.05	138.96
	3	181.98	168.91	167.86	166.78	161.96	160.73	156.72	155.89	148.92	136.64
E	1	196.32	185.23	180.98	176.43	173.75	170.53	167.15	167.54	158.46	145.45
	2	194.46	187.37	181.56	177.22	170.23	168.74	168.43	166.34	160.60	143.32
	3	196.95	185.34	183.76	177.95	169.50	169.89	168.39	164.90	160.31	143.53
F	1	201.23	194.25	189.45	184.56	180.03	176.44	174.67	173.74	167.76	150.32
	2	204.35	191.23	187.56	186.43	178.98	175.75	175.32	171.24	165.48	149.87
	3	205.04	192.77	187.41	189.11	175.20	177.25	175.13	173.69	166.86	150.68
G	1	222.33	206.41	198.89	199.43	195.04	189.67	185.47	186.32	178.65	172.43
	2	218.25	205.49	200.21	197.15	192.25	191.75	186.54	184.54	178.54	175.47
	3	224.85	211.26	204.20	199.37	193.99	191.22	188.54	186.24	182.63	172.48
H	1	232.78	221.67	218.65	211.15	204.36	203.14	201.18	201.56	191.43	179.83
	2	232.33	224.25	216.65	210.55	206.47	202.65	200.67	197.45	189.24	178.56
	3	230.80	223.65	218.28	210.28	204.77	203.75	205.11	203.63	190.86	176.87
I	1	260.85	244.54	235.21	235.85	231.24	225.76	220.05	216.43	209.44	197.34
	2	258.75	245.85	235.68	236.78	234.43	223.55	218.65	215.42	208.42	198.54
	3	259.38	249.35	239.48	237.32	234.35	222.69	218.57	215.04	211.33	200.19

**Table 2:** Temperature of the TEMs during the combustion of B10.

Operation point	Test	Temperature (°C)									
		1	2	3	4	5	6	7	8	9	10
A	1	129.34	126.32	120.05	120.54	116.55	111.97	114.32	111.97	106.64	99.45
	2	128.87	125.43	119.43	119.65	115.54	112.32	112.53	112.32	105.64	98.65
	3	125.28	127.87	123.31	115.67	118.16	115.61	113.05	111.71	108.60	102.50
B	1	164.05	152.32	146.67	144.56	139.74	137.05	136.87	134.56	131.42	127.65
	2	165.54	155.67	147.89	144.32	138.45	136.76	138.45	134.23	128.32	128.43
	3	162.74	153.68	150.70	147.74	143.31	139.92	137.54	137.17	131.97	131.73
C	1	186.43	171.35	166.03	163.54	157.76	155.43	150.86	152.56	145.64	141.67
	2	185.54	169.87	164.53	165.32	159.76	155.64	152.32	150.76	147.68	142.21
	3	184.20	170.97	164.77	163.83	157.86	157.89	156.72	152.26	145.40	145.84
D	1	190.43	181.21	175.32	173.14	166.87	164.34	157.45	158.43	152.34	153.13
	2	192.14	180.64	173.52	171.23	165.13	166.54	158.75	156.87	151.34	150.05
	3	192.65	183.16	173.28	172.56	166.36	164.12	163.17	158.46	154.96	150.27
E	1	207.14	197.87	186.43	185.53	177.54	176.43	173.34	171.32	167.84	161.34
	2	209.43	195.36	190.54	187.43	176.46	175.54	169.43	168.64	165.43	163.34
	3	207.88	195.82	187.90	187.59	181.92	178.79	175.03	172.23	165.51	165.49
F	1	217.54	210.34	199.55	201.43	191.45	186.54	184.64	182.83	177.74	170.51
	2	219.43	208.54	198.54	200.14	190.35	183.56	182.84	178.36	176.21	168.35
	3	219.43	209.89	204.76	197.83	192.55	186.55	183.56	181.21	179.81	169.85
G	1	235.54	218.53	209.42	212.32	207.48	199.52	197.21	191.32	186.43	183.05
	2	236.32	220.84	210.83	210.75	210.31	200.86	199.53	192.34	187.53	183.54
	3	240.67	217.90	214.13	207.86	207.08	205.50	191.44	196.75	189.59	181.42
H	1	258.85	243.56	237.43	236.78	230.04	221.43	216.53	211.24	208.56	202.24
	2	260.43	242.54	236.64	234.54	226.89	223.14	218.94	208.84	208.65	198.75
	3	258.89	243.71	234.14	236.02	226.23	221.76	217.48	210.85	210.27	202.31
I	1	274.53	255.43	242.76	242.76	242.68	233.45	224.84	224.67	215.54	208.43
	2	275.04	257.74	241.52	241.25	238.96	230.54	225.78	222.54	217.84	209.64
	3	272.22	255.07	244.66	243.22	241.27	230.42	227.80	222.57	214.83	211.99

**Table 3:** Temperature of the TEMs during the combustion of Diesel.

Operation point	Test	Temperature (°C)									
		1	2	3	4	5	6	7	8	9	10
A	1	114.53	110.12	104.56	105.01	102.23	99.45	101.30	98.78	98.56	94.34
	2	117.02	109.45	105.23	102.67	105.67	100.45	99.54	100.12	95.45	95.45
	3	113.96	107.79	109.80	103.72	101.34	105.02	98.89	99.96	97.08	96.98
B	1	150.21	133.65	132.32	132.21	124.37	128.32	123.32	122.34	117.45	112.34
	2	152.34	132.23	133.02	133.23	127.34	127.14	121.67	122.43	119.34	111.76
	3	153.06	135.52	134.05	133.14	128.24	127.58	123.80	123.18	117.15	112.80
C	1	168.23	158.43	157.33	151.45	150.23	149.21	144.35	138.96	131.35	124.42
	2	170.43	157.79	155.43	153.67	151.34	147.45	142.23	140.42	133.34	125.43
	3	168.76	160.99	157.10	152.65	152.33	145.57	144.43	139.54	132.21	125.87
D	1	181.23	165.42	161.34	161.78	158.21	155.75	152.53	150.32	140.45	128.45
	2	179.89	163.45	162.85	160.34	157.57	156.21	150.32	151.23	142.32	128.54
	3	179.09	165.41	162.68	163.76	156.84	156.28	150.45	150.28	143.98	127.43
E	1	191.97	182.34	181.35	172.35	170.42	165.32	165.25	163.43	155.49	140.33
	2	192.34	183.67	178.94	173.24	168.32	167.43	164.45	164.76	154.86	142.54
	3	192.38	184.49	179.74	174.34	170.12	167.29	165.96	164.02	157.11	141.45
F	1	200.15	190.45	187.45	182.23	175.47	174.46	172.23	170.24	163.45	148.56
	2	201.36	191.35	185.45	184.30	177.86	172.45	170.34	171.65	163.45	146.98
	3	198.37	189.31	185.79	184.90	176.86	173.44	172.98	171.53	164.29	148.01
G	1	210.34	205.35	196.57	193.87	184.57	183.98	183.45	181.85	173.24	158.76
	2	212.34	201.45	198.45	194.03	185.64	182.34	181.34	180.12	171.47	161.34
	3	215.57	202.11	196.61	191.22	183.86	184.72	182.80	181.30	173.51	161.85
H	1	222.24	214.54	209.35	200.23	196.42	196.86	193.45	191.23	181.34	168.43
	2	218.34	214.56	207.98	198.45	195.48	194.97	191.82	192.75	184.21	170.32
	3	223.38	217.34	208.11	203.96	192.17	193.11	189.29	192.74	183.12	170.92
I	1	250.54	237.67	232.56	227.54	215.34	214.03	213.42	208.43	202.74	185.01
	2	252.74	235.63	231.34	225.63	214.53	212.13	210.34	210.87	202.86	182.84
	3	252.00	236.20	227.03	225.67	215.73	214.25	214.49	208.18	205.47	183.19

**Table 4:** Pressure drop in the TEG.

Operation Points	P_drop_ (kPa)								
	B5			B10			Diesel		
	Test 1	Test 2	Test 3	Test 1	Test 2	Test 3	Test 1	Test 2	Test 3
A	0.45	0.45	0.45	0.41	0.41	0.41	0.54	0.54	0.54
B	0.66	0.66	0.66	0.58	0.58	0.59	0.80	0.80	0.80
C	0.74	0.74	0.74	0.67	0.67	0.67	0.86	0.86	0.86
D	0.81	0.81	0.81	0.73	0.73	0.73	0.89	0.89	0.89
E	0.86	0.86	0.86	0.81	0.81	0.81	0.98	0.98	0.98
F	0.90	0.90	0.90	0.85	0.84	0.85	1.00	1.00	1.00
G	0.96	0.96	0.97	0.90	0.90	0.90	1.05	1.05	1.05
H	1.00	1.00	1.00	0.93	0.93	0.93	1.07	1.07	1.07
I	1.08	1.08	1.08	1.00	1.00	1.00	1.20	1.20	1.20

Table 5 shows the power output of the TEG during the experiments, while Table 6 shows the power loss resulting from the pressure drop introduced by the TEG in the engine exhaust system. Table 7 shows the exhaust emissions (i.e., the emissions of CO, CO_2_, NO, NO_X_, HC, and smoke opacity) of the engine while operating with TEG and without TEG for the use of B5. Likewise, Table 8 shows the exhaust emissions of the engine while operating with TEG and without TEG, for the use of B10. Similarly, Table 9 shows the exhaust emissions of the engine while operating with TEG and without TEG, for the use of diesel during the experiments.

**Table 5:** Power output of the TEG.

Operation points	Power output (W)								
	B5			B10			Diesel		
	Test 1	Test 2	Test 3	Test 1	Test 2	Test 3	Test 1	Test 2	Test 3
A	7.43	7.37	7.46	8.81	8.75	8.84	5.51	5.52	5.54
B	16.31	16.23	16.24	17.05	17.09	17.31	13.18	13.22	13.30
C	22.73	22.70	22.73	23.35	23.38	23.47	19.27	19.32	19.34
D	26.96	27.02	26.90	27.61	27.50	27.72	23.40	23.36	23.41
E	33.70	33.63	33.68	34.76	34.70	35.02	30.16	30.21	30.32
F	37.39	37.26	37.40	41.45	41.10	41.51	33.84	33.85	33.86
G	42.06	41.96	42.49	49.42	49.71	49.70	38.24	38.17	38.27
H	50.36	50.19	50.41	64.63	64.42	64.48	43.66	43.54	43.66
I	65.27	65.25	65.59	71.18	71.06	71.15	57.99	57.77	57.85

**Table 6:** Power loss of the TEG.

Operation points	Power loss (W)								
	B5			B10			Diesel		
	Test 1	Test 2	Test 3	Test 1	Test 2	Test 3	Test 1	Test 2	Test 3
A	5.25	5.21	5.27	5.61	5.58	5.64	4.85	4.86	4.87
B	7.55	7.51	7.52	7.92	7.94	8.04	7.06	7.08	7.13
C	8.82	8.81	8.82	9.22	9.23	9.27	8.38	8.40	8.41
D	9.56	9.58	9.54	9.95	9.91	9.99	9.18	9.17	9.19
E	10.65	10.63	10.64	10.92	10.90	11.00	10.31	10.32	10.36
F	11.19	11.15	11.20	11.50	11.40	11.51	10.89	10.89	10.89
G	11.83	11.80	11.95	12.13	12.20	12.20	11.53	11.51	11.54
H	12.91	12.87	12.92	13.55	13.51	13.52	12.28	12.25	12.28
I	14.62	14.61	14.69	15.28	15.25	15.27	14.04	13.99	14.01

**Table 7:** Emissions B5.

Emissions (g/kWh)	Operation points		A	B	C	D	E	F	G	H	I
CO (g/kWh)	Test 1	T	0.81	0.91	0.95	1.03	1.39	2.29	2.47	3.46	5.19
		WT	0.83	0.94	0.98	1.06	1.42	2.35	2.54	3.56	5.34
	Test 2	T	0.80	0.93	0.96	1.02	1.42	2.24	2.41	3.53	5.24
		WT	0.82	0.96	0.99	1.05	1.46	2.30	2.48	3.63	5.39
	Test 3	T	0.83	0.92	0.97	1.04	1.40	2.26	2.43	3.48	5.29
		WT	0.85	0.95	1.00	1.07	1.44	2.32	2.50	3.58	5.44
CO_2_ (g/kWh)	Test 1	T	102.49	135.23	143.58	173.49	179.82	183.82	230.68	249.92	367.35
		WT	105.27	138.91	147.49	178.20	184.72	188.83	236.96	256.73	377.35
	Test 2	T	101.45	138.90	145.30	171.02	183.83	179.99	225.41	254.57	370.48
		WT	104.21	142.69	149.26	175.67	188.84	184.90	231.55	261.50	380.56
	Test 3	T	105.01	136.57	147.26	175.25	181.54	181.37	227.22	251.50	374.36
		WT	107.86	140.29	151.27	180.01	186.48	186.31	233.41	258.35	384.55
NO (g/kWh)	Test 1	T	0.85	0.98	1.11	1.49	1.64	1.73	1.84	1.89	2.09
		WT	0.87	1.00	1.13	1.53	1.68	1.77	1.88	1.93	2.14
	Test 2	T	0.84	1.00	1.12	1.47	1.68	1.69	1.80	1.93	2.11
		WT	0.86	1.02	1.14	1.51	1.72	1.73	1.84	1.97	2.16
	Test 3	T	0.87	0.99	1.13	1.51	1.66	1.71	1.82	1.91	2.13
		WT	0.89	1.01	1.15	1.55	1.70	1.75	1.86	1.95	2.18
NOx (g/kWh)	Test 1	T	1.13	1.18	1.43	1.91	1.99	2.09	2.30	2.29	2.60
		WT	1.16	1.21	1.47	1.96	2.05	2.16	2.36	2.36	2.68
	Test 2	T	1.12	1.21	1.45	1.89	2.03	2.05	2.25	2.33	2.62
		WT	1.15	1.25	1.49	1.93	2.09	2.12	2.31	2.40	2.70
	Test 3	T	1.16	1.19	1.47	1.93	2.01	2.07	2.26	2.31	2.65
		WT	1.19	1.23	1.51	1.98	2.07	2.14	2.32	2.38	2.73
HC (g/kWh)	Test 1	T	0.03	0.03	0.03	0.05	0.05	0.05	0.05	0.06	0.06
		WT	0.03	0.03	0.04	0.05	0.05	0.05	0.06	0.06	0.06
	Test 2	T	0.03	0.03	0.03	0.05	0.05	0.05	0.05	0.06	0.06
		WT	0.03	0.03	0.04	0.05	0.05	0.05	0.06	0.06	0.06
	Test 3	T	0.03	0.03	0.04	0.05	0.05	0.05	0.05	0.06	0.06
		WT	0.03	0.03	0.04	0.05	0.05	0.05	0.06	0.06	0.07
Smoke opacity (HSU)	Test 1	T	4.08	4.29	5.64	6.45	8.49	10.75	12.05	19.74	24.12
		WT	4.19	4.41	5.79	6.62	8.73	11.05	12.38	20.27	24.77
	Test 2	T	4.04	4.40	5.71	6.36	8.68	10.53	11.78	20.10	24.32
		WT	4.15	4.53	5.86	6.52	8.92	10.82	12.09	20.65	24.98
	Test 3	T	4.18	4.33	5.78	6.51	8.57	10.61	11.87	19.86	24.58
		WT	4.29	4.45	5.94	6.69	8.81	10.90	12.19	20.40	25.24

**Table 8:** Emissions B10.

Emissions (g/kWh)	Operation points		A	B	C	D	E	F	G	H	I
CO (g/kWh)	Test 1	T	0.49	0.57	0.62	0.74	1.14	1.68	2.14	2.95	4.43
		WT	0.50	0.59	0.64	0.77	1.18	1.73	2.21	3.05	4.57
	Test 2	T	0.48	0.58	0.64	0.73	1.13	1.68	2.13	3.00	4.46
		WT	0.49	0.60	0.66	0.76	1.17	1.73	2.20	3.10	4.60
	Test 3	T	0.47	0.59	0.63	0.72	1.15	1.71	2.09	2.97	4.50
		WT	0.48	0.61	0.65	0.75	1.20	1.76	2.16	3.07	4.64
CO_2_ (g/kWh)	Test 1	T	88.22	119.90	126.22	155.32	163.88	166.66	214.64	233.73	334.26
		WT	91.59	123.62	130.12	160.12	168.95	171.82	221.28	240.96	344.60
	Test 2	T	86.29	121.87	129.67	152.92	162.36	166.99	213.02	237.43	336.48
		WT	89.59	125.64	133.69	157.65	167.39	172.15	219.60	244.77	346.89
	Test 3	T	85.32	123.99	127.99	151.69	166.27	169.75	209.72	235.04	339.66
		WT	88.58	127.84	131.95	156.38	171.41	175.00	216.21	242.31	350.17
NO (g/kWh)	Test 1	T	0.60	0.86	0.90	1.31	1.49	1.58	1.68	1.76	1.88
		WT	0.62	0.89	0.93	1.36	1.55	1.64	1.74	1.83	1.95
	Test 2	T	0.59	0.87	0.92	1.29	1.47	1.58	1.66	1.79	1.89
		WT	0.61	0.90	0.95	1.34	1.53	1.64	1.72	1.86	1.96
	Test 3	T	0.58	0.88	0.91	1.28	1.51	1.61	1.64	1.77	1.91
		WT	0.60	0.92	0.94	1.33	1.57	1.67	1.70	1.84	1.98
NOx (g/kWh)	Test 1	T	1.32	1.28	1.55	2.01	2.08	2.30	2.38	2.40	2.88
		WT	1.36	1.32	1.59	2.07	2.14	2.38	2.45	2.47	2.97
	Test 2	T	1.30	1.30	1.59	1.97	2.06	2.31	2.37	2.44	2.90
		WT	1.34	1.34	1.63	2.03	2.12	2.39	2.44	2.51	2.99
	Test 3	T	1.28	1.32	1.57	1.96	2.11	2.35	2.33	2.42	2.92
		WT	1.32	1.36	1.61	2.02	2.17	2.43	2.40	2.49	3.02
HC (g/kWh)	Test 1	T	0.02	0.02	0.03	0.04	0.04	0.04	0.05	0.05	0.06
		WT	0.02	0.02	0.03	0.04	0.04	0.05	0.05	0.05	0.06
	Test 2	T	0.02	0.02	0.03	0.04	0.04	0.04	0.05	0.05	0.06
		WT	0.02	0.02	0.03	0.04	0.04	0.05	0.05	0.05	0.06
	Test 3	T	0.02	0.02	0.03	0.04	0.04	0.04	0.05	0.05	0.06
		WT	0.02	0.02	0.03	0.04	0.05	0.05	0.05	0.05	0.06
Smoke opacity (HSU)	Test 1	T	2.10	2.58	3.50	5.26	6.62	8.63	10.19	17.67	21.20
		WT	2.16	2.66	3.61	5.42	6.83	8.90	10.51	18.22	21.85
	Test 2	T	2.05	2.62	3.60	5.18	6.56	8.65	10.12	17.95	21.34
		WT	2.11	2.70	3.71	5.34	6.76	8.92	10.43	18.51	22.00
	Test 3	T	2.03	2.66	3.55	5.14	6.71	8.79	9.96	17.77	21.54
		WT	2.09	2.75	3.66	5.29	6.93	9.06	10.27	18.32	22.21

**Table 9:** Emissions diesel.

Emissions (g/kWh)	Operation points		A	B	C	D	E	F	G	H	I
CO (g/kWh)	Test 1	T	1.18	1.34	1.56	1.69	1.77	2.96	3.15	4.61	6.22
		WT	1.19	1.37	1.60	1.73	1.82	3.04	3.24	4.72	6.37
	Test 2	T	1.20	1.32	1.54	1.65	1.79	2.93	3.12	4.58	6.15
		WT	1.22	1.35	1.58	1.69	1.84	3.01	3.20	4.69	6.30
	Test 3	T	1.22	1.36	1.52	1.67	1.81	2.99	3.09	4.67	6.12
		WT	1.24	1.39	1.56	1.71	1.86	3.07	3.17	4.78	6.26
CO_2_ (g/kWh)	Test 1	T	113.89	155.94	162.87	196.72	191.70	195.41	242.38	260.94	411.23
		WT	116.71	159.81	166.90	201.60	196.45	200.26	248.38	267.41	421.42
	Test 2	T	116.34	153.07	160.78	191.87	194.24	193.17	239.48	259.00	406.81
		WT	119.23	156.86	164.76	196.63	199.05	197.96	245.42	265.42	416.90
	Test 3	T	118.61	158.50	158.25	194.00	196.66	197.59	237.39	264.02	404.66
		WT	121.54	162.43	162.17	198.81	201.53	202.50	243.27	270.57	414.69
NO (g/kWh)	Test 1	T	1.03	1.05	1.28	1.62	1.66	1.87	1.92	1.95	2.36
		WT	1.05	1.07	1.31	1.65	1.69	1.91	1.96	1.99	2.41
	Test 2	T	1.05	1.03	1.26	1.58	1.68	1.85	1.90	1.93	2.34
		WT	1.07	1.05	1.29	1.61	1.71	1.89	1.94	1.97	2.39
	Test 3	T	1.07	1.07	1.24	1.60	1.70	1.89	1.88	1.97	2.32
		WT	1.09	1.09	1.27	1.63	1.73	1.93	1.92	2.01	2.37
NOx (g/kWh)	Test 1	T	0.91	0.96	1.23	1.66	1.81	1.86	2.04	2.11	2.38
		WT	0.94	0.99	1.27	1.71	1.86	1.91	2.10	2.17	2.45
	Test 2	T	0.93	0.94	1.21	1.62	1.83	1.84	2.02	2.09	2.36
		WT	0.96	0.97	1.25	1.67	1.88	1.89	2.08	2.15	2.43
	Test 3	T	0.95	0.98	1.19	1.64	1.85	1.88	2.00	2.13	2.34
		WT	0.98	1.01	1.23	1.69	1.90	1.93	2.06	2.19	2.41
HC (g/kWh)	Test 1	T	0.03	0.03	0.04	0.05	0.05	0.06	0.06	0.06	0.07
		WT	0.03	0.03	0.04	0.05	0.05	0.06	0.06	0.06	0.07
	Test 2	T	0.03	0.03	0.04	0.05	0.05	0.06	0.06	0.06	0.07
		WT	0.03	0.03	0.04	0.05	0.05	0.06	0.06	0.06	0.07
	Test 3	T	0.03	0.03	0.04	0.05	0.05	0.06	0.06	0.06	0.07
		WT	0.03	0.03	0.04	0.05	0.05	0.06	0.06	0.06	0.07
Smoke opacity (HSU)	Test 1	T	5.51	6.35	7.64	9.25	9.36	12.11	13.04	22.73	27.56
		WT	5.57	6.43	7.76	9.41	9.57	12.38	13.36	23.29	28.23
	Test 2	T	5.63	6.24	7.55	9.02	9.48	11.97	12.89	22.56	27.26
		WT	5.69	6.32	7.66	9.18	9.69	12.24	13.21	23.11	27.93
	Test 3	T	5.74	6.46	7.43	9.12	9.60	12.25	12.77	23.00	27.12
		WT	5.80	6.54	7.54	9.28	9.81	12.52	13.09	23.56	27.78

Table 10 shows the specifications of the diesel engine (SOKAN- SK-MDF300) used during the experiments. Table 11 describes the operating points considered during the experiments, which were selected according to the engine characteristics. Finally, Table 12 shows the properties of diesel and biodiesel blends, obtained in the test laboratory.

**Table 10:** Specifications of the diesel engine.

Engine: SOKAN, SK-MDF300 SOKAN			
Engine type	1 cylinder, 4 Strokes	Maximum power	4.6 hp at 3600 rpm
Bore x stroke	78 × 62.57 mm	Intake system	Naturally Aspirated
Displaced volume	299 CC	Injection system	Direct injection
Compression ratio	20:1	Injection Angle	20° BTDC

**Table 11:** Experimental operating points.

Operating points	Engine torque (Nm)	Rotation speed (rpm)
A	3.0	3000
B		3400
C		3800
D	4.5	3000
E		3400
F		3800
G	6.0	3000
H		3400
I		3800

**Table 12:** Physicochemical properties of the fuels.

Property	Units	Standards	Diesel	B5	B10
Density	kg/m^3^	ASTM D1298	821.5	823.1	827.5
Viscosity	cSt	ASTM D445	2.64	2.65	2.66
Flash point	°C	ASTM D93	76	85	96
Cloud point	°C	ASTM D2500	6.5	7.2	8.3
Pour point	°C	ASTM D97	3.1	3.5	3.8
NHV	MJ/kg	ASTM D240	44.05	43.89	43.25

## 2 Experimental design, materials, and methods

Fig. 1 shows the test bench where the experiments were developed and measurements of the different operational parameters. The test bench includes an alternator to measure the engine's power output and three data acquisition systems (DAQ) to control and measure the engine and TEG parameters during the experiments. A cooling system was used on the surface of the TEG to control the surface temperature. Moreover, to measure the emissions of CO_2_, NO_X,_ and HC, a gas analyzer (BrainBee AGS-688, electromagnetic class E2) was used, while a Bacharach (PCA 400) gas analyzer was used to measure NO and CO. In addition, an opacimeter (BrainBee OPA-100) was used to measure smoke opacity in the exhaust.

A diesel engine (SOKAN- SK-MDF300) was used during the experiments (see Table 10).

According to the engine characteristics, nine operating points were selected to evaluate the most representative operating points (see Table 11). The experimental design was developed with a multilevel factorial design whit three levels (i.e., minimum, medium, and maximum) for the input variables (i.e., rotation speed, torque, and fuel). The measures on each operating point were repeated three times.

The TEG used in the experiments includes a heat exchanger, 20 thermoelectric modules (TEMs), and a cooling system to control the surface temperature.

The TEMs are located over the thermal surfaces of the heat exchanger, on a thin layer of thermal paste used to enhance the heat conduction, and compensate for the mechanical tolerances in the device. Thermoelectric modules 1 to 10, located in the upper surface, are symmetrical with TEMs 11 to 20 and yield the same generation of electricity.

The energy conversion efficiency (η) of the TEG, understood as the ratio between the power output $\left({\text{P}}_{\text{output}}\right)$ and the heat input was calculated as:

(1) $$\text{η}=\frac{{\text{P}}_{\text{output}}}{\dot{\text{m}}\cdot {\text{c}}_{\text{p}}\cdot \left({\text{T}}_{\text{in}}-{\text{T}}_{\text{out}}\right)}$$

where $\dot{\text{m}}$ is the exhaust flow, ${\text{c}}_{\text{p}}$ is the specific heat of the exhaust, ${\text{T}}_{\text{in}}$ is the exhaust input temperature, and ${\text{T}}_{\text{out}}$ is the exhaust output temperature.

The properties of diesel and biodiesel blends were measured in a Test Laboratory, following the use of the US ASTM standard tests. The properties of these fuels for each test are shown in Table 12.
